# *SUPERMAN* strikes again in legumes

**DOI:** 10.3389/fpls.2023.1120342

**Published:** 2023-01-30

**Authors:** Ana L. Rodas, Edelín Roque, Rim Hamza, Concepción Gómez-Mena, José Pío Beltrán, Luis A. Cañas

**Affiliations:** Instituto de Biología Molecular y Celular de Plantas (Consejo Superior de Investigaciones Científicas-Universidad Politécnica de Valencia), Ciudad Politécnica de la Innovación, Valencia, Spain

**Keywords:** SUPERMAN, legumes, Medicago truncatula, MtSUP, compound inflorescence, flower development, flower number

## Abstract

The *SUPERMAN* (*SUP*) gene was described in *Arabidopsis thaliana* over 30 years ago. *SUP* was classified as a cadastral gene required to maintain the boundaries between reproductive organs, thus controlling stamen and carpel number in flowers. We summarize the information on the characterization of *SUP* orthologs in plant species other than Arabidopsis, focusing on the findings for the *MtSUP*, the ortholog in the legume *Medicago truncatula*. *M. truncatula* has been widely used as a model system to study the distinctive developmental traits of this family of plants, such as the existence of compound inflorescence and complex floral development. *MtSUP* participates in the complex genetic network controlling these developmental processes in legumes, sharing conserved functions with *SUP*. However, transcriptional divergence between *SUP* and *MtSUP* provided context-specific novel functions for a *SUPERMAN* ortholog in a legume species. *MtSUP* controls the number of flowers per inflorescence and the number of petals, stamens and carpels regulating the determinacy of ephemeral meristems that are unique in legumes. Results obtained in *M. truncatula* provided new insights to the knowledge of compound inflorescence and flower development in legumes. Since legumes are valuable crop species worldwide, with high nutritional value and important roles in sustainable agriculture and food security, new information on the genetic control of their compound inflorescence and floral development could be used for plant breeding.

## Introduction

Most angiosperm flowers are organized in four concentric whorls: sepals (W1), petals (W2), stamens (W3) and carpel/s (W4), (Smyth et al., 1990). The number of floral organs and the placement of the organs within each whorl are genetically determined and MADS-box floral homeotic genes play a crucial role in the specification of floral organ identity (Bowman et al., 2012). Other classes of genes, the ones that determine the boundaries of different cell identities, are also crucial players during floral development (Yu and Huang, 2016). They were classified as “cadastral genes”, to which the Arabidopsis *SUPERMAN* (*SUP*) gene was assigned (Bowman et al., 1992; Sakai et al., 1995).

**Figure 1:**
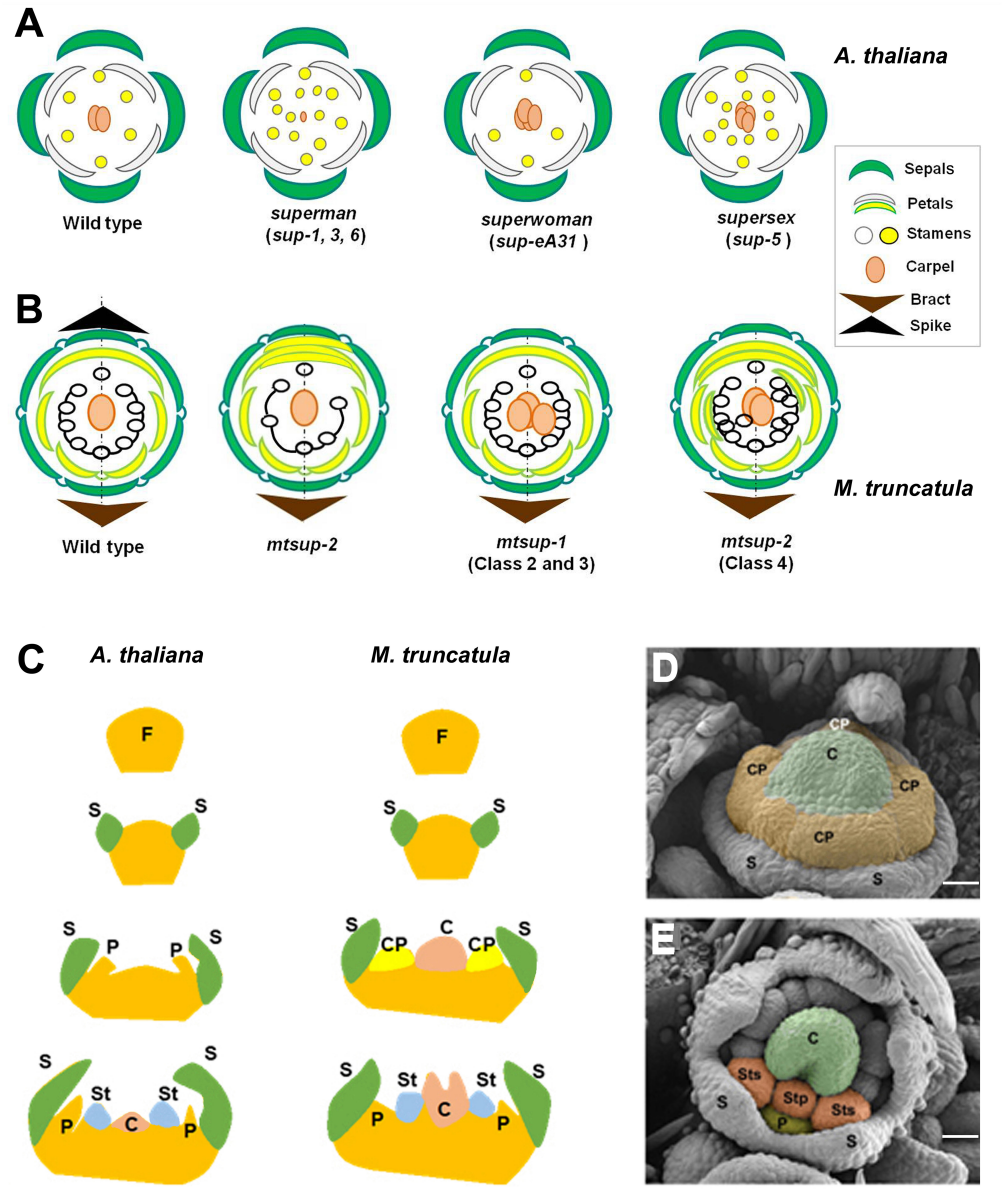
Comparative floral development of *Arabidopsis thaliana* and *Medicago truncatula*. **(A)** Comparative schematic representation among *superman* (*sup*) mutant alleles in Arabidopsis regarding floral organ number. Wild *type A. thaliana* flower: 4 sepals, 4 petals, 6 stamens and two fused carpels. **(B)** Comparative schematic representation among *superman* (*mtsup*) mutant alleles in Medicago regarding floral organ number. Wild type *M. truncatula* flower: 5 sepals, 5 petals, 10 stamens, 9 fused (staminal tube) and one free, and a single carpel. In Arabidopsis, the ‘superman’ class of mutants harbors supernumerary stamens and reduced or absent carpel, resembling *mtsup-2* showing additional petals at the expense of stamens. An increased number of carpels characterize the “superwoman” class. Similar phenotypes displayed the *mtsup-1* (class 2 and 3) alleles, with two or three carpels in *M. truncatula.* The “supersex” class, to which *sup-5* allele belong, produces more stamens and additional carpels. This phenotype is observed in *mtsup-1* allele (class 4). Also, additional petals are produced by this allele. **(C)** Left. In *A*. *thaliana* organ differentiation is centripetal and sequential. First are differentiated the sepal primordia, then the petal primordia, the stamen primordia and finally the carpel primordium. Right. In *M. truncatula*, the four common primordia differentiate petals and stamens in W2 and W3 respectively. **(D)** Floral meristem of *M. truncatula* showing the early carpel primordium (C, green) in the centre, the four common primordia (CP, orange) and the sepal primordia (S). **(E)** Each common primordium differentiates petals (P, yellow) in W2 and antepetal (Stp) and antesepal (Sts) stamens in W3 (orange). VM, vegetative meristem; FM, floral meristem; I1, primary inflorescence meristem; I2, secondary inflorescence meristem; spk, spike; S, sepal primordium; CP, common primordium; P, petal primordium; St, stamen primordium; C, carpel primordium. Scale bars, 25 μm in **(D, E)** Adapted from Benlloch et al., 2003; Breuil-Broyer et al., 2016; Prunet et al., 2017 and Rodas et al., 2021.

*SUP* is a transcriptional repressor, extensively studied in *Arabidopsis thaliana*, that encodes a plant-specific EPF-like protein with one Cys_2_-His_2_ zinc finger DNA binding domain and a C-terminus EAR-like (DLELRL) motif (Sakai et al., 1995; Hiratsu et al., 2002; Hiratsu et al., 2003; Hiratsu et al., 2004). The specific expression of *SUP* at the boundary between W3 and W4 (Sakai et al., 1995; Prunet et al., 2017) led to its classification as a cadastral gene specifying the stamens-carpel boundary. The supernumerary male organs at the expense of the female one of the *sup-1* mutant (**Figure 1A**) (Bowman et al., 1992) was initially associated with the expansion of the MADS-box genes *APETALA3* (*AP3*) and *PISTILLATA* (*PI*) expression closer to the centre of the floral meristem (Sakai et al., 1995; Prunet et al., 2017). Initially, models to explain the *SUP* function were based on a single allele: *sup-1* (*flo-10*) (Bowman et al., 1992; Sakai et al., 1995; Prunet et al., 2017). However, the study of other *sup* alleles displaying phenotypes deviating from *sup-1* (**Figure 1A**) has shed light on the *SUP* functions (Breuil-Broyer et al., 2016; Prunet et al., 2017; Xu et al., 2018). *SUP* is a gene controlling the stamens-carpel boundary setting and is linked to floral meristem termination (FMT) at the early stages of flower development.

It has been described that *SUP* is required for the correct timing to turn off *WUSCHEL* (*WUS*) from the floral meristem centre (FMC), thus controlling the floral meristem termination. *WUS* activity is required for the stem cell division at the floral meristem centre, a prolonged expression of *WUS* would lead to a delayed floral meristem termination, and more floral organs could be produced. However, *SUP* and *WUS* do not show an overlapping spatial expression and, in *sup* mutants, *WUS* expression is prolonged (Prunet et al., 2017; Xu et al., 2018). Moreover, *SUP* contributes to carpel medial region formation and the tissues derived from this region (Breuil-Broyer et al., 2016).

These studies provided new information to generate different models to explain *SUP* functions. One of the models proposes that *SUP* indirectly promotes floral meristem termination by repressing B-class genes. This model explains the different *sup* alleles phenotypes (**Figure 1A**), showing an indistinct male-female boundary and a sporadic carpel development (*sup-1*). By contrast, the increased number of stamens and carpels in the *sup-5* mutant (**Figure 1A**) supports a second model that proposes that *SUP* controls the balance of cell proliferation and differentiation at W3 and W4 (Breuil-Broyer et al., 2016). To this regard, the effect of the overexpression of *SUP*-like genes supports the activity of *SUP* as a cell proliferation control gene (Nandi et al., 2000; Bereterbide et al., 2001; Hiratsu et al., 2002; Kazama et al., 2009; Nibau et al., 2011; Zhao et al., 2014). Recent studies demonstrated that *SUP* regulates both stem cell proliferation in the floral meristem and floral organogenesis through fine-tuning auxin biosynthesis (Xu et al., 2018). This mechanism might explain all *sup* mutant phenotypes (**Figure 1A**). Studies in *SUP* have shown its broad spectrum of action, highlighting how different are the floral phenotypes according to the type of mutation (Bowman et al, 2012; Breuil-Broyer et al, 2016).

## Compound inflorescence and floral development in legumes. Distinctive traits

In addition to the well-known capacity to fix nitrogen symbiotically, some distinctive features of legumes are the presence of compound leaves and inflorescences and a complex floral development (Ferrándiz et al., 1999; Singer et al., 1999; Benlloch et al., 2003). All these traits make them of interest for the study of unique developmental processes (Hofer and Ellis, 2014; Cañas and Beltrán, 2018).

Most legumes show complex raceme inflorescences with more than one branching. In the model legume *Medicago truncatula* the primary inflorescence meristem (I1) differentiates a secondary inflorescence meristem (I2). The existence of the I2 is linked to the compound inflorescence development and is a distinctive feature compared to Arabidopsis, which produces a unique inflorescence meristem (IM) before differentiating the floral meristem (FM) (Tucker, 2003; Benlloch et al., 2007). The I2 is a transient meristem, and its identity is given by a genetic function of *VEGETATIVE1* (*VEG1*) in *Pisum sativum* and *MtFRUITFULLc* (*MtFULc*) in *M. truncatula* (Cheng et al., 2018). It has been proposed that this function was derived from the sub-functionalization of the *AGL79* MADS-box gene clade within the *AP1/SQUA/FUL* family (Berbel et al., 2012). The perpetual activity of the I2 meristem will define the number of flowers per inflorescence and its termination as a residual vegetative organ (stub or spike) in the legume compound inflorescences (Benlloch et al., 2003; Benlloch et al., 2015). In the model legume *M. truncatula*, the identity of the I1 and FM, also involved in this developmental process, are specified by *MtTERMINAL FLOWER1* (*MtTFL1*) *and MtAPETALA1* (*MtAP1*) or *MtPROLIFERATING INFLORESCENCE MERISTEM* (*MtPIM*), respectively (Benlloch et al., 2006; Cheng et al., 2018). Their spatial and temporal expression and mutual repression control the compound inflorescence development in *M. truncatula* (Cheng et al., 2018).

The wild type flower of *M. truncatula* (**Figure 1B**) displays pentamerous floral organs per whorl: five sepals in W1, five petals in W2 (a keel petal formed by two fused petals, two wing petals and one standard or vexillum), 10 stamens in W3 (nine fused in a staminal tube and one free) and a single carpel in W4 (Benlloch et al., 2003; Cañas and Beltrán, 2018). In contrast to Arabidopsis, organ differentiation shows a high degree of spatial and temporal overlapping. Even more characteristic is the presence of common primordia (CP), ephemeral meristems from which petals and stamens will differentiate, and the early carpel differentiation (Ferrándiz et al., 1999; Tucker, 2003; Benlloch et al., 2003; Roque et al., 2018). The model species *A. thaliana* (**Figure 1C** left) shows a centripetal and sequential organ differentiation. First, the sepal primordia are differentiated, then the petal primordia, followed by the stamen primordia, and finally, the carpel primordium. In contrast, *M. truncatula* (**Figure 1C** right) shows unidirectional differentiation of the organ primordia with a high degree of overlapping. Unique differences are the presence of four common primordia and the early carpel primordium differentiation (**Figures 1D, E**). Despite the functional divergence of the duplicated floral homeotic MADS-box genes in *M. truncatula*, the specification of the floral organs is conserved in this model legume (Roque et al., 2018).

## MtSUPERMAN: Conserved and new functions controlling compound inflorescence and floral development

The *SUP* gene has been widely studied in *A. thaliana*. However, there is scant information on the role of *SUP* orthologs in other plant species. The petunia *PhSUP* gene (Nakagawa et al., 2004) had been the only *SUP* ortholog functionally characterized on its own species until it was studied in the model legume *M. truncatula* (Rodas et al., 2021). Recently, the *SMALL REPRODUCTIVE ORGANS* (*SRO*) gene was described as the *SUP* ortholog in rice (Xu et al., 2022).

The results obtained from the functional characterization of *MtSUP* in Medicago (Rodas et al., 2021) uncovered new context-specific functions in a different plant species. This information may have changed not only the previously proposed idea of *SUPERMAN* as a boundary gene but also made *MtSUP* a key player of the complex regulatory network behind the compound inflorescence development, being an undescribed function for a *SUP* ortholog in eudicots. Nevertheless, there are also similarities between *MtSUP* and other *SUP* orthologs regarding flower development.

The floral phenotypes of *mtsup* mutants (**Figure 1B**) are different to Arabidopsis, Petunia and rice mutants in several respects. However, *sup*, *phsup* and *mtsup* mutants have in common the increase in the numbers of both stamens and carpels in their respective flowers. Thus, the early floral meristem function of *SUP* is conserved in these three species (Nakagawa et al., 2004; Rodas et al., 2021). However, the rice *SUP* ortholog controls the size of male and female organs but not their number (Xu et al., 2022).

**Figure 2:**
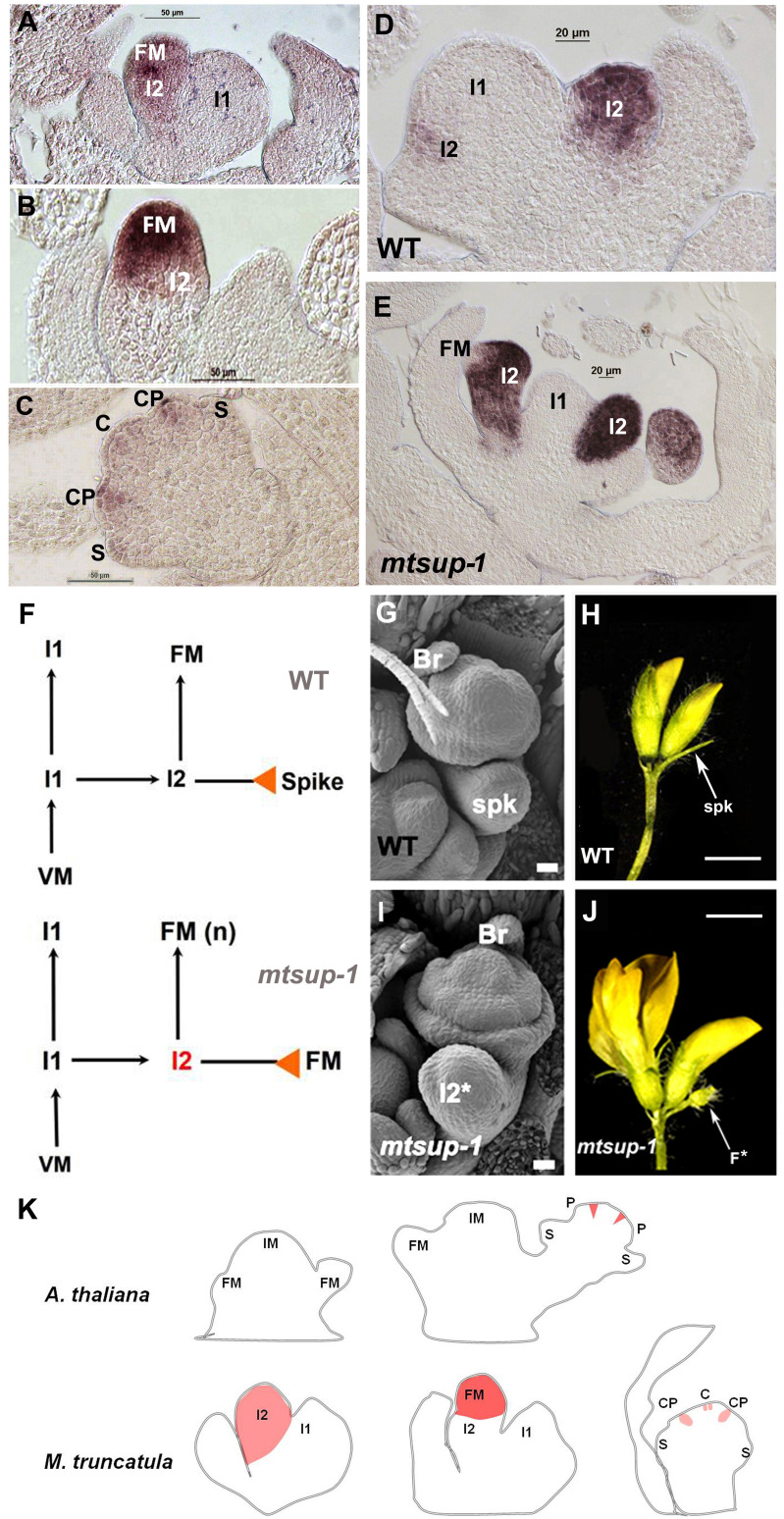
*MtSUP* controls compound inflorescence development in *M. truncatula*. **(A)** *MtSUP* transcript is firstly detected in the I2. **(B)** Later on *MtSUP* activity is detected in the FM. **(C)** During early floral development *MtSUP* expression is detected in the common primordia (CP). **(D)** *MtFULc* expression in the wild type (WT) flower. **(E)** *MtFULc* expression in the *mtsup-1* mutant. *MtFULc* transcript occupies a wider area in *MtSUP* mutants compared to the WT. **(F)** Schematic representation of the compound inflorescence development in Medicago with the formation of an I2 and the terminal spike in the WT and a new FM instead the spike in the *mtsup-1* mutant. **(G)** SEM image of a WT floral primordium with its respective bract and spike. **(H)** The WT of *M. truncatula* R108 produces one or two flowers per inflorescence and terminates in a spike. **(I)** In *mtsup-1*, the I2* (future spike) acquires floral identity. **(J)** In the *mtsup-1* mutant the residual cells of the I2 terminate as a new flower (F*) instead a spike. **(K)** Comparative schematic representation of *SUP* and *MtSUP* expression patterns during inflorescence and flower development in Arabidopsis and Medicago. *SUP* and *MtSUP* are orthologs that have functionally diverged through changes in their gene transcription patterns. IM, inflorescence meristem; I1, primary inflorescence meristem; I2, secondary inflorescence meristem; FM, floral meristem; CP, common primordium; S, sepal primordium; P, petal primordium; C, carpel primordium; Br, bract; Spk, spike; F*, new flower. Scale bars, 20 μm in **(G, I)**, and 2 mm in **(H, J)** Adapted from Rodas et al. (2021).

*MtSUP* transcript is firstly detected in the I2 meristem and later in the FM (**Figures 2A, B**). The expression pattern of *MtSUP* during floral organogenesis (Rodas et al., 2021) showed that even before the carpel primordium is initiated *MtSUP* transcript is already detected in the floral meristem centre and later in the common primordia (**Figure 2C**). The proliferation of extra petals was a distinctive feature discovered for the *mtsup* mutants during floral development (**Figure 1B**). At the common primordia, the meristematic cells that will produce petals and stamens coexist, and a given number of meristematic cells will give place to the organ primordia (Bossinger and Smyth, 1996).

As a role already described for *SUP*, *MtSUP* might control cell proliferation in the common primordia. In other words, *MtSUP* is involved in the determinacy of the common primordia as prolonged maintenance of these meristematic cells can give rise to extra organs (Bowman et al., 1989; Bossinger and Smyth, 1996). The supernumerary petals, stamens and carpels in *mtsup* mutants (**Figure 1B**) might also be explained by a delayed floral meristem termination linked to *MtWUS* persistence, as also occurs for *WUS* in *sup* mutants of *A. thaliana* (Prunet et al., 2017; Xu et al., 2018). *MtWUS* expression is not detected in the wild type after the floral apex flattens (Rodas et al., 2021). This is consistent with the early carpel initiation in legumes (Ferrándiz et al., 1999), as floral meristem termination happens when the pool of stem cells of the floral meristem centre is set to a female fate (Prunet et al., 2009). In *mtsup-1*, the expression of *MtWUS* is prolonged, thus the pool of stem cells remains undifferentiated during more time at the floral meristem centre. Contrary to *SUP*, which is not expressed in the floral meristem centre and plays a non-cell-autonomous function there (Prunet et al., 2017). *MtSUP* and *MtWUS* expression overlaps, both spatially and temporally at the I2 and the floral meristem centre, which would allow them to interact physically (Rodas et al., 2021). *MtSUP* has a novel function in the common primordia determinacy and seems to conserve its cell antiproliferative role in this unique feature of legumes.

A proper carpel primordium formation requires a correct floral meristem termination (Sakai et al., 2000; Prunet et al., 2017). *MtSUP* is expressed in the carpel marginal tissue that will develop the parietal placenta. It agrees with the defects in the marginal derived tissues of the gynoecium in *mtsup* mutants. Defects in placenta morphogenesis were also observed in the Petunia *phsup* mutants (Nakagawa et al., 2004), and the strong *sup-5* mutant (**Figure 1A**) of *A. thaliana* (Gaiser et al., 1995). Therefore, the *SUP* orthologs *PhSUP* and *MtSUP* are required for proper floral meristem termination and the correct development of the carpel marginal tissues (Nakagawa et al., 2004; Rodas et al., 2021). Common aberrancies in the development of the placenta impacted ovule development in *mtsup* mutants, reducing fertility. Similar phenotypes were reported for Arabidopsis *sup-5* (Gaiser et al., 1995) and Petunia *phsup* mutants (Nakagawa et al., 2004). Thus, the late floral function of *SUP* controlling ovule development is conserved in these three species (Gaiser et al., 1995; Nakagawa et al., 2004; Breuil-Broyer et al., 2016; Rodas et al., 2021).

Unlike Arabidopsis, *MtSUP* was first detected in the whole I2 (**Figures 2A, B**), similar to the expression of the I2 identity gene *MtFULc* (**Figures 2D, E**). This expression matched with the multiflowered phenotype in *mtsup* mutants, assigning *MtSUP* a determinant role in controlling the maturation rate of the I2. This novel function has not been described for any *SUP*-like gene. In *M. truncatula* cv.R108, the I2 derived from the I1 divides to produce one or two floral meristems (Benlloch et al., 2003). The I2 is a transient state between the vegetative and the reproductive tissue that remains immature until the floral identity acquisition (Prusinkiewicz et al., 2007). After producing a floral meristem, the remaining cells of the I2 enter senescence and produce the spike (Tucker, 1989; Benlloch et al., 2007). In *mtsup* mutants, the I2 gives rise to more floral meristems than the wild type because the residual cells of the I2 terminate as a floral meristem instead of a spike (**Figures 2F–J**). The I2 determinacy could also be linked to the gradual turn-off of *MtWUS* in the I2. As *MtWUS* prolongs its expression, the I2 could extend its activity in *mtsup* mutants, or *MtSUP* could influence the I2 activity by controlling cell proliferation. *MtFULc* transcript occupies a broader area in *mtsup* mutants compared to the wild type **(** **Figures 2D, E** **),** and there might be more cells expressing *MtFULc*. According to the expression analysis, *MtSUP* also seems to restrict *MtPIM* expression to the floral meristem and this restriction could be considered another way to control the determinacy of the I2. In *mtsup* mutants, *MtPIM* invades the expression domain of *MtFULc* in the I2 and the remnant cells that lose their vegetative nature and acquire floral identity (Rodas et al., 2021).

*SUPERMAN* was classified as a “cadastral gene” after studying the *sup-1 (flo10)* allele (Bowman et al., 1992). However, the results obtained in Medicago might support that the conserved ancestral function of *SUP*-like genes is the control of cell proliferation rather than a cadastral function. *MtSUP* does not have a typical boundary expression pattern since it is expressed in the whole I2 and floral meristem. This transcript localization correlates with the floral and inflorescence phenotypes of *mtsup-1* mutant. Thus, the model that proposes the balance of cell proliferation explains *mtsup* mutants better than the model that considers that *SUP* is related to the repression of B-class MADS-box genes to the floral meristem centre. Indeed, *MtPI* expression (Roque et al., 2018) in *mtsup-1* expands towards the W1 instead of expanding to the floral meristem centre (Rodas et al., 2021). In both flower and inflorescence development, there is no need to invoke a boundary function to explain *mtsup* mutants. Certainly, the phenotypic consequences of *sup* mutations in Arabidopsis are correlated to an over-proliferation of cells at W3 and the floral stem cells at the floral meristem centre (Prunet et al., 2009; Prunet et al., 2017; Xu et al., 2018). In addition, in *P. hybrida* the specific expression of *PhSUP* in the stamen primordia and the excessive proliferation of cells at the connective tissue in *phsup1* anthers suggest that the control of cell division and growth is the function of this *SUP* ortholog (Nakagawa et al., 2004). Similar conclusions were reached with the *SUP* ortholog in rice. The authors stated that *SRO* is not a cadastral gene based on its expression pattern and the mechanisms through which *SRO* regulates reproductive organ development (Xu et al., 2018).

From an evolutionary point of view, *SUP* and *MtSUP* are orthologs that have functionally diverged through changes in their gene transcription patterns while keeping some common functions (**Figure 2K**). Such changes can occur through transposition, rearrangement, duplication or point mutations in the regulatory regions (Carroll, 2005), which are frequent after whole-genome duplications (WGD) events, a common phenomenon in the evolution of angiosperms (Cui et al., 2006). The WGD event that pre-dated the speciation of legumes ~50–60 million years ago had an essential role in structuring the *M. truncatula* genome and in the success of papilionoid legumes (Cannon et al., 2006). However, these rounds of polyploidization have contributed mainly to the gradual decline in the conserved synteny between species, as is the case for Arabidopsis and *M. truncatula* (Young et al., 2011). An example is the absence of collinearity in the flanking regions of *MtSUP* and *SUP* in their respective genomes (Rodas et al., 2021).

The functional study of *SUP* orthologous genes in other legume species (alfalfa, common bean) or plants with complex inflorescence (*i.e.* tomato, mustard) could help to understand *SUP*-like genes implications in the development of higher order meristems (*i.e*. I2). Alternatively, they could show the emergence of new functions for transcription factors when they are expressed in species with different architectures. Recently, the *SINGLE FLOWER* (*SFL*) gene, a MYB transcription factor expressed in the I2, was shown to perform a similar role to *MtSUP* in chickpea (Caballo et al., 2022). Multiflowered pea and chickpea mutants have been reported for three decades (Murfet, 1985; Singer et al., 1999; Gaur and Gour, 2002; Srinivasan et al., 2006; Devi et al., 2018). However, the correlation between the multiflowered mutants and the genes responsible requires further studies. The genes involved in the specification and determinacy of inflorescence meristems could be used as bioengineering tools to optimize inflorescence traits (Wang et al., 2021). In line with this, *SUP*-like genes in other crops should be studied to determine their possible roles in inflorescence development.

## Author contributions

ER, AR and LC wrote the manuscript and RH, CG-M and JB contributed with valuable comments during the manuscript writing. All authors contributed to the article and approved the submitted version.
